# Molecular and Lifestyle Factors Modulating Obesity Disease

**DOI:** 10.3390/biomedicines8030046

**Published:** 2020-03-01

**Authors:** Maria Teresa Valenti, Angelo Pietrobelli, Maria Grazia Romanelli, Elia Franzolin, Giovanni Malerba, Donato Zipeto, Monica Mottes, Luca Dalle Carbonare

**Affiliations:** 1 Department of Medicine, University of Verona, 37134 Verona, Italy; elia.franzolin@univr.it (E.F.); luca.dallecarbonare@univr.it (L.D.C.); 2 Department of Surgical Sciences, Dentistry, Gynecology and Pediatrics, University of Verona, 37134 Verona, Italy; angelo.pietrobelli@univr.it; 3 Pennington Biomedical Research Center, Baton Rouge, LA 70808, USA; 4 Department of Neurosciences, Biomedicine and Movement Sciences, University of Verona, 37134 Verona, Italy; mariagrazia.romanelli@univr.it (M.G.R.); giovanni.malerba@univr.it (G.M.); donato.zipeto@univr.it (D.Z.); monica.mottes@univr.it (M.M.)

**Keywords:** obesity, mesenchymal stem cells, differentiation, diet, physical activity

## Abstract

Obesity adversely affects bone health by means of multiple mechanisms, e.g., alterations in bone-regulating hormones, inflammation, and oxidative stress. Substantial evidence supports the relationship between adiposity and bone disorders in overweight/obese individuals. It is well known that the balance between mutually exclusive differentiation of progenitor cells into osteoblasts or adipocytes is controlled by different agents, including growth factors, hormones, genetic and epigenetic factors. Furthermore, an association between vitamin D deficiency and obesity has been reported. On the other hand, regular physical activity plays a key role in weight control, in the reduction of obesity-associated risks and promotes osteogenesis. The aim of this review is to highlight relevant cellular and molecular aspects for over-weight containment. In this context, the modulation of progenitor cells during differentiation as well as the role of epigenetics and microbiota in obesity disease will be discussed. Furthermore, lifestyle changes including an optimized diet as well as targeted physical activity will be suggested as strategies for the treatment of obesity disease.

## 1. Introduction

Obesity is a serious health problem in nearly all European countries as well as in the rest of the world, and the percentage of people affected by obesity has been increasing considerably in the last four decades. Obesity disease is a complex pathology. Alterations in progenitor cells differentiation, epigenetics, genetics and environmental factors as well as lifestyle concur to the pathogenesis of obesity. In this review, we will address the role of mesenchymal stem cells (MSCs) differentiation and the mechanisms of epigenetic modifications (including microRNAs) promoting adipogenesis in order to understand their roles in obesity. In addition, we will discuss the microbiota involvement in regulating adipogenesis. Finally, we will discuss the role of physical activity in preventing obesity in order to preserve a healthy life.

## 2. Obesity and Mesenchymal Stem Cells

The differentiation process of mesenchymal stem cells plays a central role among the factors involved in obesity-related diseases. The disrupted adipo-osteogenic balance has been associated to different pathophysiological processes, such as aging and obesity and osteopenia-related disorders. MSCs are self-renewing cells that can undergo multiple alternative differentiation pathways, i.e., osteogenic, chondrogenic, adipogenic, myogenic and neurogenic [1]. Therefore, a tightly controlled alternative commitment of MSCs plays a critical role in their homeostasis maintenance. Control and regulation of MSCs differentiation outcomes have been extensively investigated.

### 2.1. Stem Cell Lineage Differentiation towards Osteogenesis or Adipogenesis

Different signalling pathways are involved in the regulation of adipogenesis and osteogenesis. Among signals that define cells fate, Wnt exhibits both pro-osteogenic and antiadipogenic activities. Wnt glycoproteins can be secreted to act as signaling molecules via interaction with their specific receptors [2]. In fact, Wnt signaling pathways are involved in different cellular processes such as proliferation, migration and stem cells self-renewal [3]. Wnt signaling can be activated either by a canonical pathway, where β-catenin protein is involved, or a non-canonical pathway, excluding the β-catenin protein involvement [2]. Signaling cascades that promote osteogenic or adipogenic differentiation of the MSC lineage generally converge on one of two key transcription factors RUNX2 and PPARγ. PPARγ (peroxisome proliferator-activated receptor gamma) is generally considered the master regulator of adipogenesis; its anti-osteoblastogenic effect has also been well described [4]. RUNX2 on the other hand, is regarded as the master regulator of osteogenesis [5]. RUNX2 activates the expression of *COL1A1* (Collagen type I isoform 1) and *COL1A2* (collagen type I isoform 2), *ALP* (alkaline phosphatase) and *OCN* (osteocalcin) genes [6]. It has been demonstrated that RUNX2 inhibits adipogenesis when overexpressed [7].

Previously we have demonstrated that in osteoporotic patients *PPARγ* expression was higher while *RUNX2* expression was lower compared to controls [8] In young male mice it has been demonstrated that high fat diet (HFD)-induced obesity affects the availability of osteoblastic progenitors in bone, to the advantage of adipogenesis [9].

### 2.2. Stem Cell Lineage Differentiation towards Chondrogenesis or Adipogenesis

The balance between chondrogenesis and osteogenesis plays as well an important role in obesity related disorders. Several studies have demonstrated that a subtle crosstalk occurs between chondrogenesis and adipogenesis. The addition of dexamethasone to human synovium-derived stem cells during chondrogenic induction promotes also adipogenesis [10]. Adipogenic features, such as signet-ring morphology, have been observed in pericytes cultured in chondrogenic medium [11]. Furthermore, in murine bone marrow-derived MSCs, deletion of *Vav Guanine Nucleotide Exchange Factor 1* (*Vav1*) promotes adipogenesis and inhibits chondrogenesis. Vav1 protein activity may lead to cytoskeletal actin transcriptional alterations and rearrangements [12] Accordingly, *Vav1* overexpression increases chondrogenic differentiation and inhibits adipogenic differentiation [13].

The transcription factors involved in chondrogenesis and adipogenesis are actually interrelated; hence their interactions affect mesenchymal stem cells commitment. Downregulation of the chondrogenic transcription factor *SOX9* occurs during adipogenesis in order to allow the expression of adipogenic transcription factors *CCAAT/enhancer-binding protein beta (C/EBPβ)* and *CCAAT/enhancer-binding protein delta (C/EBPδ*) [14]. On the contrary, upregulation of *SOX9* and downstream chondrogenic genes *COL2A1* and *ACAN* leads to the suppression of C/EBPα, C/EBPβ and C/EBPδ factors [14]. These findings demonstrated that a negative regulation between C/EBP members and SOX9 occurs. However, it must be mentioned that other studies reported how SOX9 may play a positive role in adipogenic differentiation since it stabilizes *C/EBPβ* mRNA [15]. In addition, C/EBP factors are able to transactivate *SOX9* in cultured cell lines; such a complex scenario suggests that tangled interactions occur between these transcription factors and deserve further investigations [16]. Different molecular factors, e.g., FGFs, IGF1, TGFβ, BMPs and others control the balance between chondrogenic and adipogenic differentiation of mesenchymal stem cells. FGF2 exerts a positive effect in promoting chondrogenic differentiation when supplemented during cell expansion [17]. FGF2 inhibitory effect on adipogenesis has also been observed; this effect has been shown to involve the high mobility group A-2 (HMGA2) [18]. Additionally, FGF1 reduces the expression of BMP and activin membrane-bound inhibitor homolog (BAMBI) by affecting the PI3K pathway promoting adipogenic differentiation [19]. In particular, down-regulation of the Erk1/2 pathway as well as the association to PI3K pathway are required for IGF1 effectiveness on chondrogenesis and adipogenesis [20,21]. Moreover, TGFβ and Hedgehog pathways can induce chondrogenesis and impair adipogenesis. Bone morphogenetic proteins signaling can promote either chondrogenesis or adipogenesis through the activation of Smad1/5/8 and p38 pathways [22]. Importantly, several chemical factors exert various effects on stem cells differentiation by affecting different pathways. In fact, dexamethasone induces adipogenesis by means of C/EBPα factors whereas it can inhibit adipogenesis by upregulating *RUNX2* expression [22]. Also biochemical and biophysical factors affect the crosstalk between chondrogenesis and adipogenesis through the activation of different signaling pathways. Specifically, these signals act by regulating master trascription factors such as *SOX9* for chondrogenesis or *C/EBPs* and *PPARγ* for adipogenesis [22].

### 2.3. Obesity and Osteoporosis Appear as Partenering Traits

It has been demonstrated that obesity associated factors such as alteration of bone-regulating hormones, inflammation or oxidative stress, do affect bone health [23,24,25,26,27,28]. Lifestyle changes, including a healthier diet as well as regular physical activity, are recommended for obesity treatment. Since bone marrow MSCs in adults give rise to both osteoblasts and adipocytes in bone, it has been considered that limiting adipocytes output from MSCs should benefit the osteoblasts pool, thereby alleviating osteoporosis [29]. In addition, white adipose tissue (WAT) and brown adipose tissue (BAT) play crucial roles in storing versus wasting energy, respectively. WAT functions as the body energy storage and supply center; it predominantly consists of white adipocytes and a small fraction of immune and stromal cells [30]. In adults, mature and well differentiated adipocytes are present; stored lipids define their size. Preadipocytes and adipocite progenitors also reside in WAT, along with vascular tissue. Under conditions of increased energy inflow, when adipocytes reach their highest reserve capability, preadipocytes maturation is triggered in order to host new incoming energy. [31]. BAT is densely packed with mitochondria and produces heat through an inner mitochondrial membrane-associated protein called uncoupling protein-1 (UCP1). It decouples mitochondrial oxidative phosphorylation from ATP production and dissipates chemical energy as heat, which significantly increases energy expenditure [32]. Such activity represents an adaptive thermogenesis and it appears very useful. A recent positron emission tomography (PET-CT) study demonstrated metabolically active BAT in healthy adults, while in people with obesity and aged subjects BAT mass and activity are reduced [33]. These findings have revived the suggestion of increasing BAT amount and/or activity in order to waste energy and thus treat obesity.

## 3. Epigenetics and Adipogenesis

The importance of epigenetics in modifying gene expression is nowadays getting increasing consideration. Epigenetic regulations can occur through DNA methylation, histone modifications, non-coding RNAs driven gene silencing [34].

DNA methylation, affecting especially the cytosines in CpG islands, induces epigenetic modifications resulting in gene expression regulation [35]. In fact, addition of methyl groups to the CG sites is associated with gene silencing, while hypomethylation promotes transcription [36]

DNA methylation can be influenced by nutritional factors and it plays an important role during development, in particular during the embryonic and fetal life as well as during the early phase of postnatal development [37]. Nutritional factors can affect epigenetically the expression of genes regulating fat progenitor cells and adipocytes number [38] or the expression of genes involved in food intake regulation [39].

It has been demonstrated that among the micronutrients vitamin A, and in particular its bioactive form retinoic acid (RA), modulates the methylation of genes involved in development and metabolism [40]. Arreguin et al. demonstrated that rats, supplemented with retinyl ester or β-carotene during the suckling period, showed inWAT altered methylation profiles of CpG islands of *PPARG* (a gene involved in adipogenic differentiation), *ZFP423* (a gene involved in adipogenic determination), *PCNA* (a gene involved in proliferation), and *RBP4* (a gene involved in retinol transport). In particular, in retinyl ester-treated rats hypermethylation of *RBP4* and *PPARG2* promoters was observed, along with hypomethylation of *PCNA* promoter. Conversely, in β-carotene-treated rats hypomethylation of *RBP4* and hypermethylation of *PCNA* promoters at distinct CpGs were found, while the expression of *PPARG2* was not affected. Finally, in both treated groups, *ZFP423* promoter was found to be hypomethylated, with a consequently increased gene expression [40].

It has been demonstrated that HFD modulates DNA methylation and that the dietary intake of triacylglycerols produces metabolically active free fatty acids (FFA) [41]. Recently, it has been observed that oleic acid, a fatty acid, affects the methylation of control regions for genes involved in adipogenesis such as *PPARG* and *C/EPBα*, promoting increased gene expression [42].

Histones may undergo post-translational modifications such as acetylation, phosphorylation methylation, as well as ubiquitination. These modifications modulate chromatin arrangement and transcriptional activity. Recently, it has been suggested that beige adipocytes commitment is regulated by several histone methyl-modifying enzymes, in particular by lysine demethylase 6B, euchromatic histone-lysine N-methyltransferase 1, Jumonji domain containing 1A and histone lysine demethylase 1 [43].

Non-coding RNAs, including miRNAs, have increasingly been receiving consideration as important modulators of gene expression, which can therefore affect cell physiology. Non-coding RNAs seem to play an important role in individual susceptibility to obesity. Studies based on microarray approaches have shown differential expression of many miRNAs in human adipose tissue, by comparing normal to obese individuals. However, the functional role of most single miRNAs has not been defined. MiR-103, miR-107, and miR-143, have been found to regulate adipose tissue homeostasis [44]. MiRNAs such as miR-17-5p, miR-132 and miR-21 result to be differentially regulated in white adipose tissue of obese subjects compared to lean subjects. [45,46]. MiRNAs modulation has also been correlated with anthropometric parameters (e.g., BMI, glycaemia, insulin levels) [47]; it has also been found that specific miRNAs are downregulated in WAT of obese patients [48]. So far it has not been possible to define a specific miRNA pattern to be responsible for promoting obesity, yet miRNAs may represent good biomarkers for clinical use [49]. Circulating miRNA levels can be correlated with biochemical/metabolic/anthropometric parameters; their potential role as biomarkers for diabetes has been proposed [50]. MiRNAs can be found not only in body fluids but also in extracellular vesicles such as exosomes, released by all cell types, including adipocytes. Exosomes represent relevant tools for cell-cell communications, which may influence tissue functions [51]. Experiments in animal models demonstrated how treatment with exosomes isolated from obese mice, induced glucose intolerance and insulin resistance in lean mice. Obesity-associated exosomal miRNAs were then identified [52].

Other non-coding RNAs, such as long (>200 nt) non-coding RNAs, (lncRNAs) have been investigated as important actors in cell biology. Some of them may exert regulatory functions in adipogenesis. Lnc-BATE1 role, in particular, has been highlighted in the control of brown adipocytes development [53].

Finally, non-coding RNAs, in particular miRNAs, are regarded as potential therapeutic agents/targets. In fact, altered miRNA patterns associated with pathological conditions, may be restored by means of miRNA agonists (mimics) and antagonists (inhibitors). Similarly, abnormal expression of lncRNAs can also be knocked down. At present no specific miRNA therapies aiming at reducing fat mass in obese subjects are available, but research is this field is very active [54,55].

Therefore, it seems clear that the epigenetic regulation and control of adipogenesis play an important role in obesity disease; certainly further studies are required to clarify in depth the pathogenetic mechanisms.

## 4. Vitamin D and Obesity

Vitamin D, a lipophilic hormone involved in bone metabolism, acts by binding its receptor (VDR) which is present within the cells of most human tissues. Bone is the main reservoir of calcium and phosphorus. Vitamin D regulates calcium and phosphorus homeostasis by targeting intestine (stimulation of calcium and phosphorus absorption), kidney (induction of calcium and phosphorus resorption together with PTH) and bone (where vitamin D stimulates the stored skeletal calcium mobilization) [56].

Besides affecting bone homeostasis, Vitamin D plays an important role in the immune system development, brain development; it is also an important regulator of lungs growth. Vitamin D role is also fundamental in the prevention or treatment of degenerative diseases often associated to obesity such as insulin resistance and type 2 diabetes, cardiovascular diseases and cancer [57].

Exposure to sunlight and diet are the only sources of inactive vitamin D [58]. Its activation involves complex processes such as the conversion by hepatic vitamin D-25-hydroxylase to 25-hydroxyvitamin D [25(OH)D], which in turn undergoes an hydroxylation process to become the biologically active form 1,25-dihydroxyvitamin D [1,25(OH)D] in the kidneys [58].

Vitamin D deficiency has been observed in individuals with obesity [59]. Different factors or mechanisms have been proposed to contribute to vitamin D deficiency in obesity. Limited outdoor activity together with a poor dietary vitamin D intake have been suggested as causes [58]. However, studies in humans and in animal models have suggested fat deposits to induce vitamin D abduction in individuals with obesity [60,61]. Reduced vitamin D levels might be also a consequence of its volumetric dilution in the adipose tissue stores [62].

MSCs committed to the adipocytic lineage express VDR; in vitro experiments have demonstrated that the receptor knock-down inhibits adipogenesis [63]. Recently, it has been demonstrated that VDR affects adipose tissue remodeling by regulating energy metabolism [64]. The authors found that mice overexpressing VDR have higher levels of serum triglyceride and cholesterol compared to normal mice. In addition, VDR overexpression negatively modulates the expression of UCP1, a protein influencing the thermogenic capacity of BAT [65].

In a study involving 22 patients we observed that their waist circumference (WC) correlated with the relationship between vitamin D absorption and fat mass [66]. In particular, we suggested that adipose tissue decrease, evaluated by waist circumference measurement, can drive mesenchymal stem cells differentiation towards osteogenesis. It is noteworthy that we found an association between a surrogate measurement of visceral adiposity (i.e., waist) and vitamin D. Specific attention should be paid when body weight, BMI, and WC increase over time in patients with obesity with deficient 25(OH)D serum concentration, regardless of dietary vitamin D intake [67]. Moschonis and colleagues have recently found a significant association of vitamin D insufficiency with insulin resistance, possibly independent of obesity [68]. However, despite the above assumptions, further studies are required to assess the relationship between hypovitaminosis D and obesity.

## 5. Physical Exercise and Changes in Gut Microbiota in Obese Individuals

Body composition is affected not only by the dietary regimen, but also by physical activity. Several studies underline the role of exercise effects on mesenchymal stem cells fate [69,70,71,72]. The World Health Organization (WHO) has highlighted how sedentary life, in addition to hypertension, tobacco use and hyperglycaemia, contributes to overall mortality [73]. On the contrary, regular physical activity counteracts degenerative diseases such as cardiovascular diseases, diabetes and cancer [74]. Regular physical exercise may control body weight and ultimately contributes to obesity prevention. In most cases fatness and fitness can be considered antithetical terms [75,76,77]: fatness hampers fitness since it is very difficult, for an obese person to do the same amount of exercise as a normal-weight person. Effective weight loss may be reached by combining physical activity and restrained food intake. Notably, the onset of chronic diseases such as obesity and type II diabetes is matched with perturbations (dysbiosis) in gut microbiota [78]. Human gut microbiota is composed by trillions of symbiotic microorganisms which play important roles in maintaining intestinal homeostasis and modulating the immune system. Physical exercise can restore intestinal health favoring beneficial modifications of gut microbiota [79,80]. It has been shown that exercise contributes to increase microbial diversity in the presence of HFD, reduces inflammation and increases antioxidant enzymes [81]. Exercise-induced weight loss in turn is also responsible for changes in gut microbiota: remarkable divergences can be found in obese compared to non-obese individuals [82].

Gut microbiota is involved in the modulation of host energy metabolism. In fact, it promotes the production of short chain fatty acids which represent an alternative energy substrate for gluconeogenesis occurring in the liver [83], and it affects the hepatic production of triglycerides as well as the metabolism of lipids and carbohydrates [84].

In the gut several bacteria synthesize vitamins such as vitamin K, folic acid or thiamine [80]. Recently, we found that exercise increased the levels of two metabolites of vitamin B6 salvage pathway (pyridoxal 5′-phosphate, pyridoxamine 5′-phosphate) [85]. As vitamin B6 cannot be produced by mammals, it may be introduced in the intestine only through the diet or by symbiotic bacteria [86]. Therefore, we have suggested that increased levels of vitamin B6 might be due to the physical performance [85]. This finding is intriguing in consideration of vitamin B6 role in diabetes, a disease frequently associated to obesity. In fact, it has been suggested that vitamin B6 can affect insulin resistance by regulating adipogenesis-associated genes [87]. However, it has also been proposed that reduced levels of vitamin B6 may induce insulin resistance by increasing homocysteine levels in consequence of the disrupted activity of cystathionine-β-synthase (CBS) and cystathionine-γ-lyase (CGL), enzymes requiring vitamin B6 as coenzyme [88].

Different guidelines suggest types and frequency of exercise [89,90,91,92,93]. It has also been suggested that even low intensity activities promote health benefits [94]. Regular training can repress specific pathways involved in adipogenesis and bone resorption [95]. It has been observed that resistance exercise interferes with adipogenesis in oestrogen-deficient rats [96]. Mechanical loading signals can induce osteogenic and chondrogenic genes expression to the detriment of adipogenesis by activating the Wnt–β-catenin pathway [97]. Recently, we demonstrated that physical activity promotes the expression of important molecules such as the osteogenic determinant *RUNX2* and of chondrogenic determinant *SOX9* in circulating progenitors of male runners after a half marathon [98]. We also observed a reduced expression of the adipogenic determinant *PPARG2* in these circulating progenitors after the half marathon [98]. However, many other genes have been suggested to influence adipogenic differentiation. Recently, it has been demonstrated that the overexpression of *GNPDA2, SEMA3G, HSPA12A*, increases adipogenesis; overexpression of *SIRT1* and *SIRT2*, on the contrary, downregulates it [99].

In muscle physical exercise induces the expression of PGC1α, a transcriptional coactivator involved in mitochondrial biogenesis as well as in oxidative metabolism, which in turn stimulates the expression and regulates *Fndc5* gene [100]. *Fndc5* gene expression produces a type I membrane protein that, after an enzymatic proteolysis, can be released in peripheral blood and is called irisin. Li and coworkers demonstrated that irisin impairs adipogenesis in favor of osteogenesis in visceral fat tissue [101]. In particular, the authors demonstrated that irisin enhances mitochondrial energy metabolism in visceral adipocytes. In addition, irisin induces, in subcutaneous white adipose tissue, the expression of transcriptional regulators related to beige adipocytes such as UCP-1, PRDM16, TMEM26, CD137 and also PGC1α. All these factors produce an increased energy expenditure and counteract obesity–associated insulin resistance [101]. Moreover, by adding irisin to osteoblasts during in vitro differentiation, the authors observed the upregulation of *RUNX2, OSTERIX, OSTEOPONTIN* and enhanced mineralization.

MiRNAs play an important role in the differentiation of mesenchymal stem cells [102]. Recently, by analyzing the effects of male runners’ sera addition to cultured MSCs, we observed an increased expression of osteo-miRNAs miR21, miR129-5p, miR378 [103]. Furthermore, we found that miR188-5p expression was instead downregulated during the differentiation process of MSCs treated with runners’ sera [103]. Most likely miR188-5p takes an action in promoting the adipogenesis switch against osteogenesis [104].

## 6. Body Composition Alterations and Socio-economic Impact

The postnatal onset of obesity is highly associated with the excessive consumption of a high-caloric, high-fat diet (HFD) and reduced physical exercise. Knowledge of body composition is relevant to many disciplines. Assessment of nutritional status, tracking the course of the disease, individuals growth and aging, work conditions, are a few paradigms for which evaluation of body composition may contribute to the understanding physiological processes and may help in the treatment of a complex disease such as obesity [105]. Significant physical changes occur during the years spanning infancy through adulthood, both externally and internally. Clinicians and researchers have long reported that individuals of the same age, height, and weight [thus, same Body Mass Index (BMI)], can have different body shapes, body proportion and body composition, energy requirements, and metabolic profiles [106].

Body composition in aging is characterized by an increase in fat mass and decrease in lean tissues, including skeletal muscle mass which in elderly adults is related to reduced muscle strength and functional capability, as well as greater morbidity and mortality [107]. Increased fat mass and body fat distribution are considered important contributors to obesity-related health risks, including type 2 diabetes, cardiovascular disease, morbidity and mortality [108]. Hyperglycaemia during pregnancy can increase an offspring birth weight as well as the risk of obesity in the phase of the childhood. However, influences of maternal body composition on offspring fat mass and fat-free mass (FFM) are still unclear, with some studies finding a positive association with fat mass and others with FFM [109].

A rapid rise in obesity and being overweight due to nutrition alteration (e.g., fast food) and sedentary lifestyle have been affecting people’s health in a rapid and unprecedented way. Obesity is a very complex and multi-factorial trait. Body weight can be influenced by environmental conditions, genetic and epigenetic factors, excessive food intake not balanced by energy consumption [110]. Environmental factors can modify the relationship between obesity and adiposity genetic risk; the association strength of obesity-related genes with BMI increases in obesogenic environments.

### 6.1. Prenatal and Post-Natal Changes

Maternal pre-pregnancy BMI and gestational weight gain have been both found to be positively and independently associated with neonatal and infant adiposity [111]. Such association appears not only pre-pregnancy, but also during the postnatal stages. Lawlor and colleagues showed, in a cohort of 146,894 participants, that maternal weight gain was positively associated with offspring BMI at age 18 in siblings from women with overweight and obesity ([112].

Gene variants associated with obesity may be responsible for excessive adiposity in children. Breastfeeding, formula feeding, rapid infant growth, macronutrient intake during infancy, complementary feeding, sleep duration, screen activities all are related with obesity risk [113]. Substantial increases in prevalence developed countries have been recorded [114].

### 6.2. Adulthood Changes

Considering that the first 1000 days of life represent the most important period for preventing non-communicable diseases [115], adulthood is associated with an increase in white adipose abdominal tissue (AT) which significantly enhances insulin resistance [116]. High levels of sedentary behavior are associated with a 112% increase in the hazard ratio of diabetes, 147% increase in the risk of cardiovascular disease, 90% increase in the risk of cardiovascular mortality and 49% increase in the risk of all-cause mortality [117].

### 6.3. Senility Changes

Changes in the elderly’s lifestyle, at the time of retirement, can cause a state of chronic positive energy balance, leading to excessive accumulation of adipose tissue, a condition that accelerates the development of age-related diseases. It is becoming apparent that the obese state leads to reduced life span and body health consequences, which are similar to those found in advanced ageing. Since fat is usually the largest organ in humans, age-related changes in adipose tissue function may result in profound systemic changes. It is increasingly evident that obesity leads to health complications and reduced lifespan [118].

### 6.4. Socioeconomic Impact of Obesity

Values of BMI between 30 to 35, BMI between 35 to 40 and BMI > 40 have been associated with 25%, 50% and 100%, respectively, higher medical expenses than normal weight respectively. Sharifi and colleagues recently pointed out that among subjects in the 6-12 years age range, over 10 years the intervention would reach two million children with obesity and would cost $239 million or $119 per child reached and $237 per unit change in BMI [119]. Looking at the European perspective, a recent report showed that, due to obesity, life is becoming five to ten years shorter. Recently Konnopka et al. conducted a systematic review of illness costs studies for overweight and adiposity in Germany [120]. The pooled relative cost-differences for studies conducted won adults were +22% for the difference between normal weight and overweight and +53% for the difference between normal weight and subjects with obesity. Extrapolation of relative pooled costs-differences from bottom-up studies in the German population yielded direct and indirect costs of 22.2 billion Euros for overweight and 23.0 billion Euros for obesity [120]. Obesity can be realistically considered a public health crisis since it severely impairs people’s health and quality of life and burdens considerably national health-care budgets [121].

## 7. Conclusions

**Figure 1:**
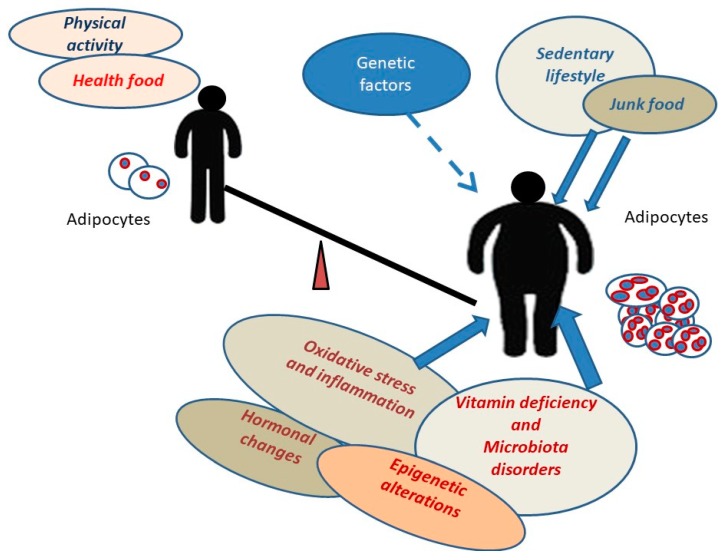
Different factors such as junk food and sedentary lifestyle together with factors promoting adipogenic differentiation of progenitor cells contribute to obesity.

Increased fat mass and body fat distribution are considered important contributors to obesity-related health risks, including type 2 diabetes, cardiovascular disease, and mortality. In addition, the socio-economic impact of obesity is considerable in our era. Various lifestyle factors as well as genetic and metabolic alterations contribute to obesity (Figure 1).

In fact, behavioral and environmental factors inducing cellular and molecular perturbations related to adipogenesis play an important role in the pathogenesis of obesity in children and adolescents and during ageing. Adipocytes originate from multipotent MSCs; strategies addressing MSCs differentiation towards alternative cell lineages may represent a promising therapeutic challenge. Different studies have identified the molecular pathways involved in MSCs’ fate choice (Table 1); it seems therefore possible to control their commitment with the goal of maintaining tissue homeostasis. Further research in this challenging field is needed. In conclusion, along with a healthy lifestyle, further research efforts should be made in order to identify proper molecular targets for counteracting, obesity associated morbidity and mortality.

## Figures and Tables

**Table 1:** Summary of the discussed topics concerning MSCs and adipogenesis.

Author	Topic	Reference
Lefterova et al.	PPARgamma and adipogenesis (2014)	[4]
Valenti et al.	Ox-PAPCs and Differentiation of MSCs (2011)	[8]
Qu et al.	Vav1 and MSCs (2016)	[13]
Stockl et al.	SOX 9 and MSC (2013)	[15]
Boney et al.	Shc and preadipocytes (2000)	[20]
Zhou et al.	Chondrogenesis versus adipogenesis (2019)	[22]
Liang et al.	Maternal HFD and Brown tissue (2016)	[122]
Maredziak et al.	Physical activity and adipogenesis (2015)	[71]
Liang et al.	Maternal obesity and epigenetics (2016)	[38]

## Endnote

We term exercise a specific form of physical activity. In particular, any movement inducing muscle contraction can be considered physical activity. However, planned physical activity aimed to improve fitness or health benefits can be defined exercise.

## Abbreviation

ALP	Alkaline Phosphatase
AT	Adipose Tissue
BAT	Brown Adipose Tissue
BMI	Body Mass Index
BMPs	Bone Morphogenic Proteins
COL1A2	Collagen type I isoform 2
D [25(OH)D	25-hydroxyvitamin DErk Extracellular signal-regulated kinase
FFM	Fat-free mass
FGFs	Fibroblast Growth Factors
GNPDA2	Glucosamine-6-Phosphate Deaminase 2
HFD	High-Fat Diet
HSPA12A	Heat Shock Protein Family A member 12A
IGF1	Insulin like growth factor 1
MSCs	Mesenchymal Stem Cells
OCN =	OsteocalcinPET-CT positron emission tomography
PI3K	PhosphatidylInositol 3-Kinase
PCNA	Proliferating Cell Nuclear Antigen
PPARγ	Peroxisome Proliferator-Activated Receptor gamma
PTH	Parathyroid Hormone
RBP4	Retinol-Binding Protein
SEMA3G	Semaphorin-3G
SIRT	Sirtuin
SMAD	Small Mother Against Decapentaplegic
TGFβ	Transforming Growth Factor-β
UCP1	Uncoupling Protein-1
VDR	Vitamin D Receptor
ZFP423	Zinc Finger Protein 423
WAT	White Adipose Tissue
WC	Waist Circumference
